# Supplementary material for: A fault-intrusion-tolerant system and deadline-aware algorithm for scheduling scientific workflow in the cloud
Source: PeerJ Comput Sci. 2021 Nov 2;7:e747. doi: 10.7717/peerj-cs.747 (PMC8576554; doi:10.7717/peerj-cs.747)
Supplement: Supplemental Information 6 [file peerj-cs-07-747-s006.zip › Myproject1.1/docs/org/workflowsim/WorkflowParser.html]

WorkflowParser


JavaScript is disabled on your browser.


- Overview
- Package
- Class
- Use
- Tree
- Deprecated
- Index
- Help

- Prev Class
- Next Class

- Frames
- No Frames

- All Classes

- Summary:
- Nested |
- Field |
- Constr |
- Method

- Detail:
- Field |
- Constr |
- Method


org.workflowsim

## Class WorkflowParser

- java.lang.Object
- - org.workflowsim.WorkflowParser

- ---

    

  ```
  public class WorkflowParser
  extends java.lang.Object
  ```

  WorkflowParser parse a DAX into tasks so that WorkflowSim can manage them

  Since:
  :   WorkflowSim Toolkit 1.0

- - ### Field Summary

    Fields

    | Modifier and Type | Field and Description |
    | --- | --- |
    | `protected java.util.Map<java.lang.String,java.lang.Double>` | `mName2Runtime` Map from task name to task runtime. |
    | `protected java.util.Map<java.lang.String,java.lang.Double>` | `mName2Size` Map from file name (data) to its size. |
    | `protected java.util.Map<java.lang.String,Task>` | `mName2Task` Map from task name to task. |
  - ### Constructor Summary

    Constructors

    Constructor and Description

    `WorkflowParser(int userId)`

    Initialize a WorkflowParser

    `WorkflowParser(int userId,
    java.lang.String fileSizePath,
    java.lang.String runtimePath,
    java.lang.String daxPath)`
  - ### Method Summary

    Methods

    | Modifier and Type | Method and Description |
    | --- | --- |
    | `java.util.List<Task>` | `getTaskList()` Gets the task list |
    | `void` | `parse()` Start to parse a workflow which includes text files and xml files. |
    | `protected void` | `setTaskList(java.util.List<Task> taskList)` Sets the task list |

    - ### Methods inherited from class java.lang.Object

      `clone, equals, finalize, getClass, hashCode, notify, notifyAll, toString, wait, wait, wait`

- - ### Field Detail


    - #### mName2Task

      ```
      protected java.util.Map<java.lang.String,Task> mName2Task
      ```

      Map from task name to task.


    - #### mName2Runtime

      ```
      protected java.util.Map<java.lang.String,java.lang.Double> mName2Runtime
      ```

      Map from task name to task runtime.


    - #### mName2Size

      ```
      protected java.util.Map<java.lang.String,java.lang.Double> mName2Size
      ```

      Map from file name (data) to its size.
  - ### Constructor Detail


    - #### WorkflowParser

      ```
      public WorkflowParser(int userId)
      ```

      Initialize a WorkflowParser

      Parameters:
      :   `userId` - the user id. Currently we have just checked single user
          mode


    - #### WorkflowParser

      ```
      public WorkflowParser(int userId,
                    java.lang.String fileSizePath,
                    java.lang.String runtimePath,
                    java.lang.String daxPath)
      ```
  - ### Method Detail


    - #### getTaskList

      ```
      public java.util.List<Task> getTaskList()
      ```

      Gets the task list

      Returns:
      :   the task list


    - #### setTaskList

      ```
      protected void setTaskList(java.util.List<Task> taskList)
      ```

      Sets the task list

      Parameters:
      :   `taskList` - the task list


    - #### parse

      ```
      public void parse()
      ```

      Start to parse a workflow which includes text files and xml files.


- Overview
- Package
- Class
- Use
- Tree
- Deprecated
- Index
- Help

- Prev Class
- Next Class

- Frames
- No Frames

- All Classes

- Summary:
- Nested |
- Field |
- Constr |
- Method

- Detail:
- Field |
- Constr |
- Method
